# Perceptions and Experiences of the Foster Family Program Among Alumni and Undergraduate Medical Students at CMC Ludhiana: An Exploratory Mixed-Methods Study

**DOI:** 10.7759/cureus.116154

**Published:** 2026-09-12

**Authors:** Pallavi Abhilasha, Abhilasha Williams, Ranjit Injety, Shereen Varghese, Pamela A Jeyaraj, Jeyaraj D Pandian

**Affiliations:** 1 Department of Psychiatry, Christian Medical College and Hospital, Ludhiana, IND; 2 Department of Dermatology, Christian Medical College and Hospital, Ludhiana, IND; 3 Department of Neurology, Christian Medical College and Hospital, Ludhiana, IND; 4 Department of Microbiology, Christian Medical College and Hospital, Ludhiana, IND; 5 Department of Radiation Oncology, Christian Medical College and Hospital, Ludhiana, IND

**Keywords:** foster family, mental health, mentor–mentee relationships, mixed method study, peer support

## Abstract

Background: Mentorship plays a crucial role in the holistic development of medical students. The Foster Family Program (FFP) at Christian Medical College (CMC), Ludhiana, is a unique mentorship model that provides academic, emotional, social, and spiritual support through a family-like structure involving faculty mentors (“foster parents”) and students (“foster siblings”).

Objective: To describe and explore foster parents’ and foster siblings’ retrospective perceptions of the FFP, including reported support, engagement, relationship continuity, strengths, challenges, and suggestions for improvement.

Methods: A mixed-methods study was conducted among MBBS alumni of CMC Ludhiana (batches 1960-2015), including foster parents and foster siblings. Quantitative data were collected via a structured online questionnaire using snowball sampling and analyzed using descriptive and inferential statistics. Qualitative data were obtained through open-ended responses and analyzed using thematic analysis. Key domains assessed included academic performance, mental health, interpersonal relationships, and career development.

Results: A total of 227 participants were included, comprising 24 (10.6%) foster parents and 203 (89.4%) foster siblings. The mean age of foster parents was 49.6 years, and that of foster siblings was 21.2 years. Most participants reported positive outcomes, with 15 (65.2%) rating the program highly. Interpersonal relationships were strengthened in 20 (87.0%) of the foster parents, while 14 (60.9%) discussed mental health concerns with their fosters. 120 (61.2%) students reported receiving support during stressful periods, and 109 (55.3%) identified their mentor as inspirational. Long-term relationships persisted, with 17 (77.3%) between foster parents and students, while 13 (6.6%) kept in touch with their foster parents only and 91 (46%) with both foster parents and siblings maintaining contact after graduation. Qualitative analysis revealed key strengths, including emotional support, academic and spiritual guidance, a safe space for sharing, and community bonding. Reported challenges included inactivity of some foster families, time constraints, financial burden, mismatched expectations, and interpersonal discomfort.

Conclusion: The FFP was associated with perceived improvements in the academic, psychosocial, and emotional well-being of medical students. While overall perceptions were positive, variability in engagement highlights the need for improved mentor-mentee matching and more structured interaction. The model demonstrates potential for replication, although contextual adaptation may be necessary.

## Introduction

The medical curriculum in India is unique in many respects and is generally considered one of the toughest programs, unlike anything a new medical student experiences. To ease students' transition into this challenging environment, medical educators of yesteryear introduced a unique fostering program to support students' educational and social development. Mentors typically possess advanced education, expertise, and professional experience [1], whereas mentees are usually in the early stages of their careers, focusing on developing specific skills and often looking to mentors as trusted role models with prior experience or familiarity with their developmental goals [2]. There is no single framework to ensure effective mentoring; instead, the framework is relative to the environment, the intention, and the individuals [3]. The foster family program (FFP) is a unique initiative offered by the Christian Medical College & Hospital, Ludhiana, which was started in 1960 to provide new medical students with a family within the college where they can share their joys, sorrows, and confusions, and find a welcoming home where they can be themselves. It is a non-competitive, supportive environment that fosters growth and nurtures students when they need it.

There are invaluable benefits to outlining a mentoring process, making the relationship life-changing. Effective mentoring requires a systematic approach that dictates direction, guidance, and cadence [4]. A mentoring relationship can therefore be viewed as a social partnership involving an “interpersonal exchange influenced by both the mentor’s and the mentee’s perceptions of each other”, and it may occur either formally or informally [5]. Formal mentoring relationships often last between 6 and 12 months, whereas those that develop informally may continue for five years or more [6]. Kram et al further describe the mentoring relationship as an intense interpersonal bond expressed through both career-related functions (such as guidance and exposure) and psychosocial support (such as counseling and role-modeling). Moreover, it is typically dyadic, professional, and intentional in nature, promoting learning and collaboration with the aim of fostering both professional and personal development [7].

Mentoring relationships do not exist in isolation; rather, they are shaped by the wider work environment in which they take place. The interaction between mentor and mentee occurs within a specific interpersonal context at the workplace and is guided by a particular developmental purpose. In this sense, the workplace sets the boundaries and influences the nature of the relational dynamics between the mentor and the mentee. From an interpersonal perspective, mentoring relationships promote collaboration through the sharing of knowledge and the transfer of workplace skills. At the same time, on an intrapersonal level, both the mentor and the mentee experience personal growth and development through the mentoring process [8].

The FFP addresses the transition of students from a protected home environment to the demanding and unfamiliar setting of medical college. Students are assigned to foster families, providing mentorship, peer support from senior and junior batches, and guidance in academics, professionalism, and personal adjustment. This system creates a safe and informal space to address challenges, prevent maladaptive behaviors, and ensure well-being. It also facilitates continued parental involvement and support when needed. Overall, the program fosters long-term bonds and provides a strong support network that extends beyond medical training. The National Medical Commission (NMC) has made it mandatory for each medical college to have a mentorship program to guide the students as they progress from being novice learners to competent graduates. As part of the CBME, a structured mentor-mentee relationship is emphasized by introducing the topic in the foundation course to help medical students understand the importance of interpersonal relationships within a health care team [9].

The FFP was established at CMC Ludhiana in 1960 as a family-oriented support system for medical students. Although it shares elements with formal mentoring approaches, it predates the Competency-Based Medical Education framework introduced in India in 2019. The present study does not evaluate the causal effectiveness or impact of the FFP. Instead, it aims to describe and explore retrospective perceptions and experiences of foster parents and foster siblings across academic, personal, social, spiritual, and relational domains, including perceived strengths, challenges, and views on the transferability of the model.

## Materials and methods

Study design and setting

This was a single-center, exploratory, cross-sectional convergent mixed-methods survey conducted among individuals associated with the FFP at Christian Medical College, Ludhiana. Quantitative closed-ended items and qualitative open-ended responses were collected concurrently through a single online questionnaire. The two strands were analyzed separately and integrated during interpretation to identify areas of convergence, complementarity, and divergence.

Participants and recruitment

Eligible participants were MBBS alumni from CMC Ludhiana graduating between 1960 and 2015 and individuals who had served as foster parents in the FFP. Participants were recruited through the alumni association, class representatives, email, social-media groups, and participant referral. The survey link was initially circulated to class representatives, who were asked to share it with eligible alumni in their respective networks. Respondents were also invited to forward the link to other eligible participants.

This was a non-probability snowball sample. No formal a priori sample-size calculation was performed because the study was exploratory and there was no reliable sampling frame or denominator for all eligible former FFP participants. As recruitment depended on network dissemination and voluntary response, a conventional response rate could not be calculated. The sample should therefore be interpreted as a self-selected group of respondents and not as representative of all FFP participants.

Questionnaire development

Because no validated instrument was available for this institution-specific, family-based mentorship model, a study-specific questionnaire was developed from program objectives and domains identified by the research team. The questionnaire was reviewed by five senior faculty members and Medical Education Unit members for relevance, clarity, comprehensiveness, and appropriateness to the study objectives. Items were revised before administration.

The instrument included demographic items, duration and nature of FFP involvement, frequency of interaction, types of support, perceptions of approachability and communication, relationship continuity, and views on usefulness and feasibility of replication. Role-specific items were used for foster parents and foster siblings. The questionnaire included closed-ended categorical and Likert-type items, along with open-ended questions about perceived strengths, challenges, and recommendations.

The questionnaire was not designed or validated as a multi-item psychometric scale. Accordingly, formal assessment of construct validity, test-retest reliability, and internal consistency for program-level constructs was not performed. Internal-consistency statistics such as Cronbach’s alpha were not applicable to isolated factual or single-item indicators. These measurement limitations were considered when interpreting results. The full role-specific questionnaire should be provided as Supplementary Appendix 1.

Quantitative analysis

Quantitative analyses were performed using SPSS version 29. Participant characteristics and item-level responses were summarized using frequencies and percentages for categorical variables and means with standard deviations for continuous variables where appropriate. Denominators are reported for each item because of item-level missing data.

Given the exploratory design, non-probability sampling, unequal group sizes, and small foster-parent sample, quantitative analyses were primarily descriptive. Foster-parent and foster-sibling responses were presented separately because they represent distinct roles and perspectives. Inferential comparisons were not used to infer program effectiveness or causal associations. Where exploratory comparisons are retained, they should be limited to directly comparable variables, use Fisher’s exact test when expected cell counts are small, report effect sizes and 95% confidence intervals, and be interpreted cautiously as hypothesis-generating.

Composite scores and correlation analyses should be removed unless their construction, scoring, missing-data rules, and measurement justification can be fully documented.

Qualitative analysis and integration

Open-ended responses were analyzed using inductive thematic analysis. Two reviewers independently coded responses, developed preliminary codes, and grouped related codes into themes. Differences were resolved through discussion and consensus. The quantitative and qualitative findings were integrated during interpretation using a joint display that identified whether qualitative themes supported, contextualized, or diverged from the quantitative findings.

Ethics

Ethical approval was obtained from the Institutional Ethics Committee of Christian Medical College Hospital, Ludhiana (approval number 4465). Electronic informed consent was obtained before survey participation. Participation was voluntary, responses were anonymized, and confidentiality was maintained.

## Results

There were 227 participants, of whom 24 (10.6%) were foster parents (mentors) and 203 (89.4%) were foster siblings (students). The age of the participants ranged from 17 to 84 years, with an average age of approximately 33 years. The mean age of the mentors was 49.6 years (SD = 13.7), while for foster siblings, it was 21.2 years (SD = 9.7). Age distribution among foster siblings was concentrated in the 20-29 age group (n = 95, 47.3%), while foster parents were more evenly distributed across older age brackets, with the highest representation in the 40-49 age group (n = 10, 41.7%). All foster parents held postgraduate degrees (100%), while foster siblings were nearly evenly split between postgraduate (n = 102, 50.2%) and undergraduate (n = 101, 49.8%) qualifications. Employment status differed notably between the groups, where 22 (91.7%) foster parents were employed, and 2 (8.3%) were retired. Among foster siblings, 104 (51.2%) were employed, and 95 (46.8%) were students (Figure 1).

**Figure 1:**
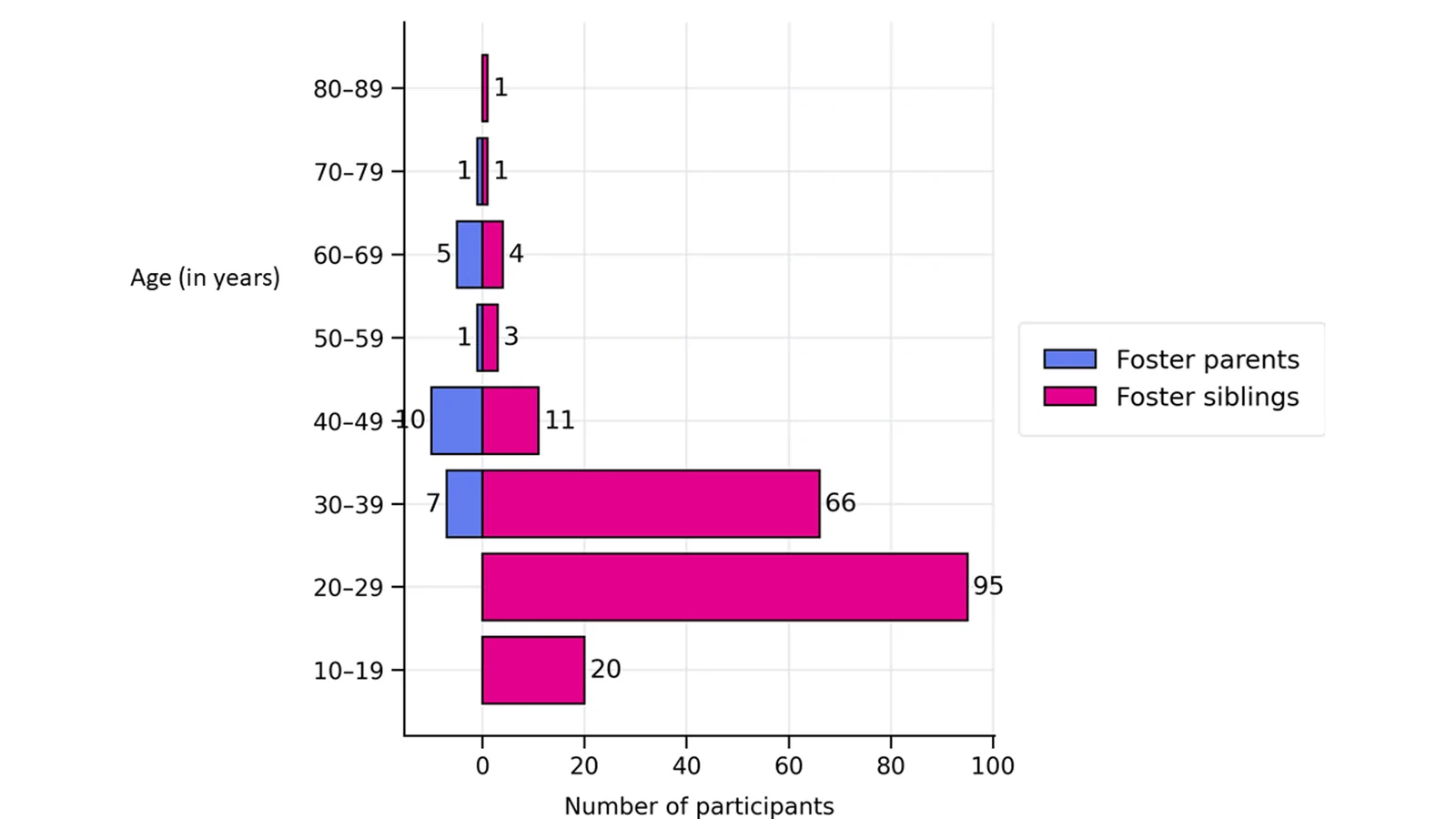
Age distribution of the participants. Foster siblings were primarily clustered in the 20–29 and 30–39 year age groups, while foster parents were mainly represented in the 30–49 year age range. The number of participants declined progressively in the older age categories

Foster parents had an average of 21.2 years of association with the institution (CMC), with 8.9 years of experience as foster parents. Foster siblings had a shorter tenure, averaging 5.8 years at CMC and 4.5 years in the FFP. The majority of foster siblings were from the batches in the 2010s (n = 89, 44.1%) and 2000s (n = 48, 23.8%), while foster parents were mostly from the batches in the 1990s (n = 9, 39.1%) and 2000s (n = 8, 34.8%). Over half of the foster parents (n = 14, 58.3%) were once foster siblings (Figure 2).

**Figure 2:**
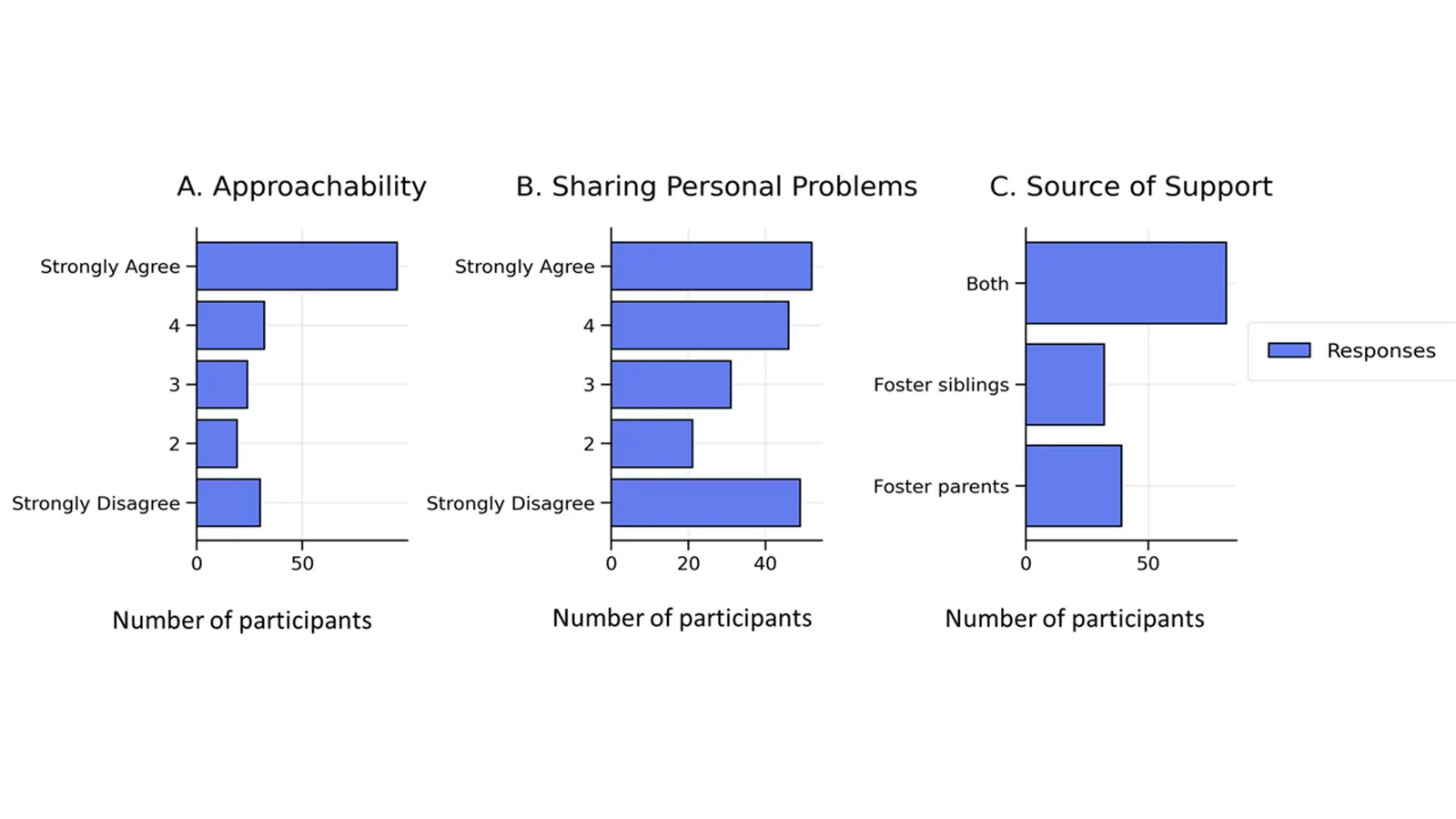
Perceived support within the FFP. (A) Most participants reported that their foster family was approachable, with the highest proportion selecting “Strongly Agree.” (B) Participants also indicated a willingness to share personal problems with their foster family, reflecting a trusting and supportive mentor–mentee relationship. (C) When asked about their primary source of support, the majority reported receiving support from both foster parents and foster siblings, highlighting the complementary role of faculty mentors and peer support within the FFP. FFP: Foster Family Program

The frequency of meetings between foster parents and siblings varied, with 84 (42.4%) foster siblings reporting quarterly meetings, 42 (21.2%) monthly, and 11 (5.6%) weekly. Notably, 61 (30.8%) could not recall the frequency of meetings. About 15 (65%) of foster parents reported that their fosters shared personal issues. Foster parents reported that their fosters shared academic performance mostly occasionally (n= 9, 39.1%), rather than regularly (monthly to quarterly) (n= 4, 17.4%), and after professional exams (n = 3, 13.0%).

Spiritual growth was acknowledged by 11 (47.8%) foster parents, while 5 (21.7%) denied such influence. A majority (n = 20, 87%) of foster parents reported that interpersonal relationships were fostered through the program. Mental health discussions occurred with 14 (60.9%) foster parents. Over half of the foster parents (n=14, 60.9%) and siblings (n = 99, 51.8%) reported that they were able to discuss academic failures with their foster families (Figure 3).

**Figure 3:**
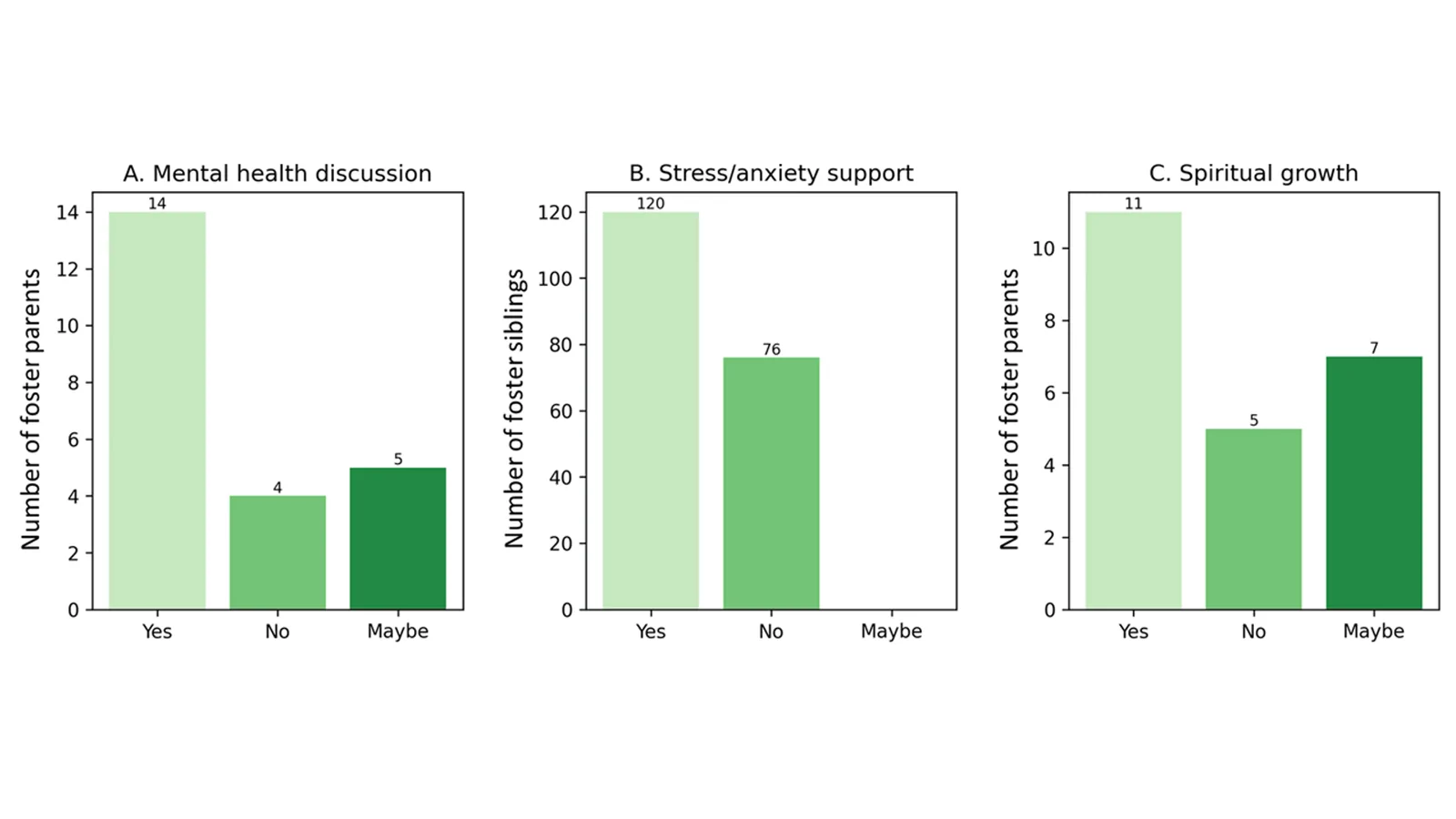
Perceived role of the FFP in emotional and spiritual support. (A) Most foster parents reported that the FFP provided opportunities for discussing mental health concerns, with the majority responding “Yes.” (B) A substantial proportion of foster siblings indicated that the program offered support during periods of stress and anxiety, highlighting its role in promoting emotional well-being. (C) Foster parents also perceived the program as contributing to students’ spiritual growth, although responses showed greater variability compared to mental health support. FFP: Foster Family Program

When asked about the benefits of the foster relationship, 15 (65.2%) of foster parents found it very beneficial, and 7 (30.4%) rated it as average. After foster siblings left college, 17 (77.3%) of foster parents reported that they maintained communication with their fosters, while foster siblings reported that 13 (6.6%) kept in touch with their foster parents only and 91 (46%) with both foster parents and siblings. Support during stressful periods was acknowledged by 120 (61.2%) foster siblings. Similarly, 103 (51.5%) reported sharing academic challenges, and 91 (51.8%) experienced academic failure. Career guidance was rated as helpful (ratings 4 or 5) by 80 (40.6%) of foster siblings. Furthermore, 109 (55.3%) agreed or strongly agreed that their mentor served as an inspiration. Continued contact at major life events was interpreted only as a descriptive indicator of relationship continuity: Six (27.3%) foster parents attended their foster child’s weddings, while 30 (19.6%) of foster siblings reported inviting their foster parents (Figure 4-5).

**Figure 4:**
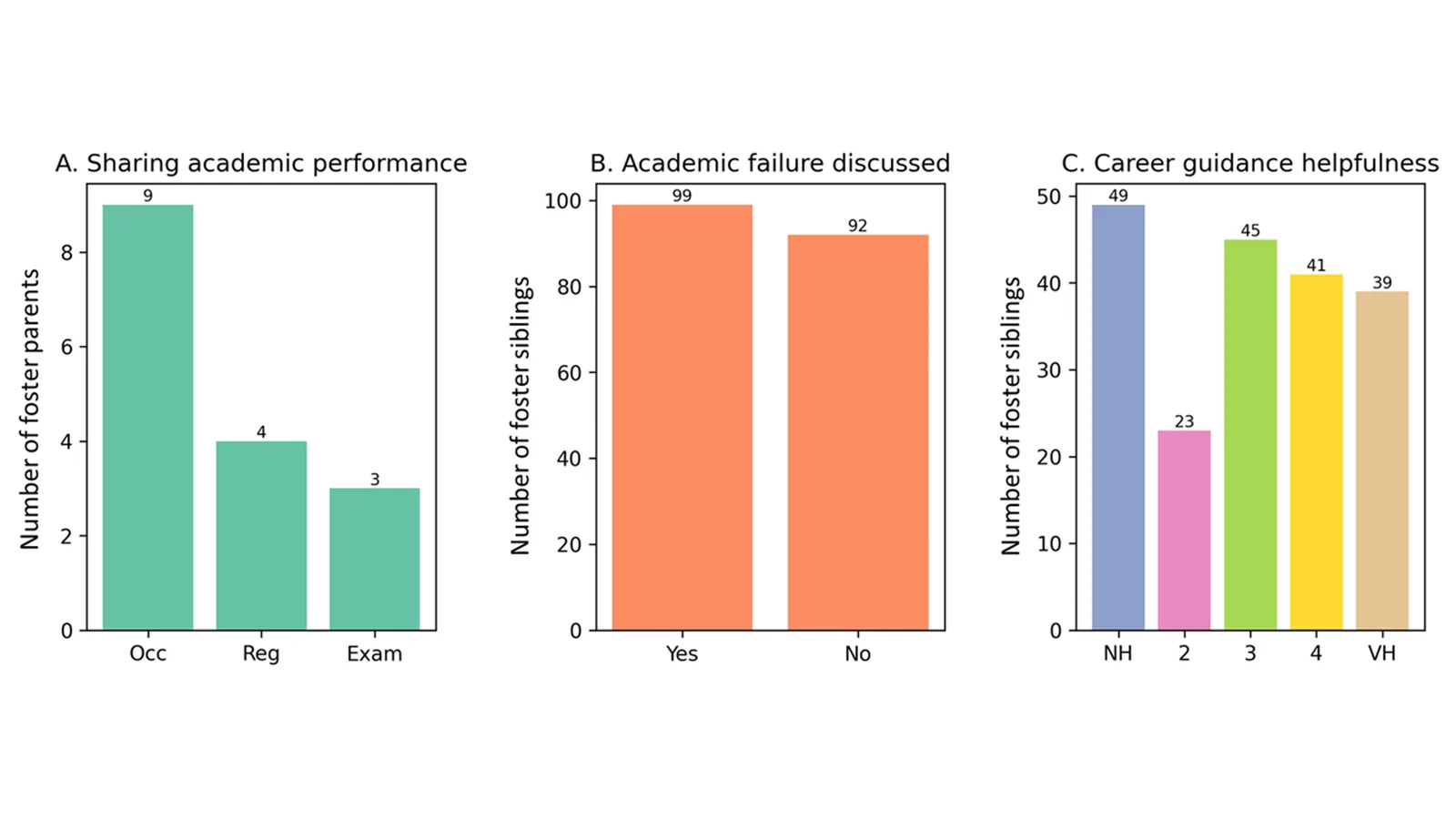
Academic support and career guidance within the FFP. (A) Foster parents reported that students most commonly shared concerns related to academic performance and attendance, followed by regular academic issues and examination-related matters. (B) Nearly equal numbers of foster siblings reported discussing academic failures with their foster families, indicating that the program provides a platform for addressing academic challenges and setbacks. (C) Foster siblings generally perceived the career guidance provided through the FFP as beneficial, with a substantial proportion rating it as moderately to very helpful. FFP: Foster Family Program

**Figure 5:**
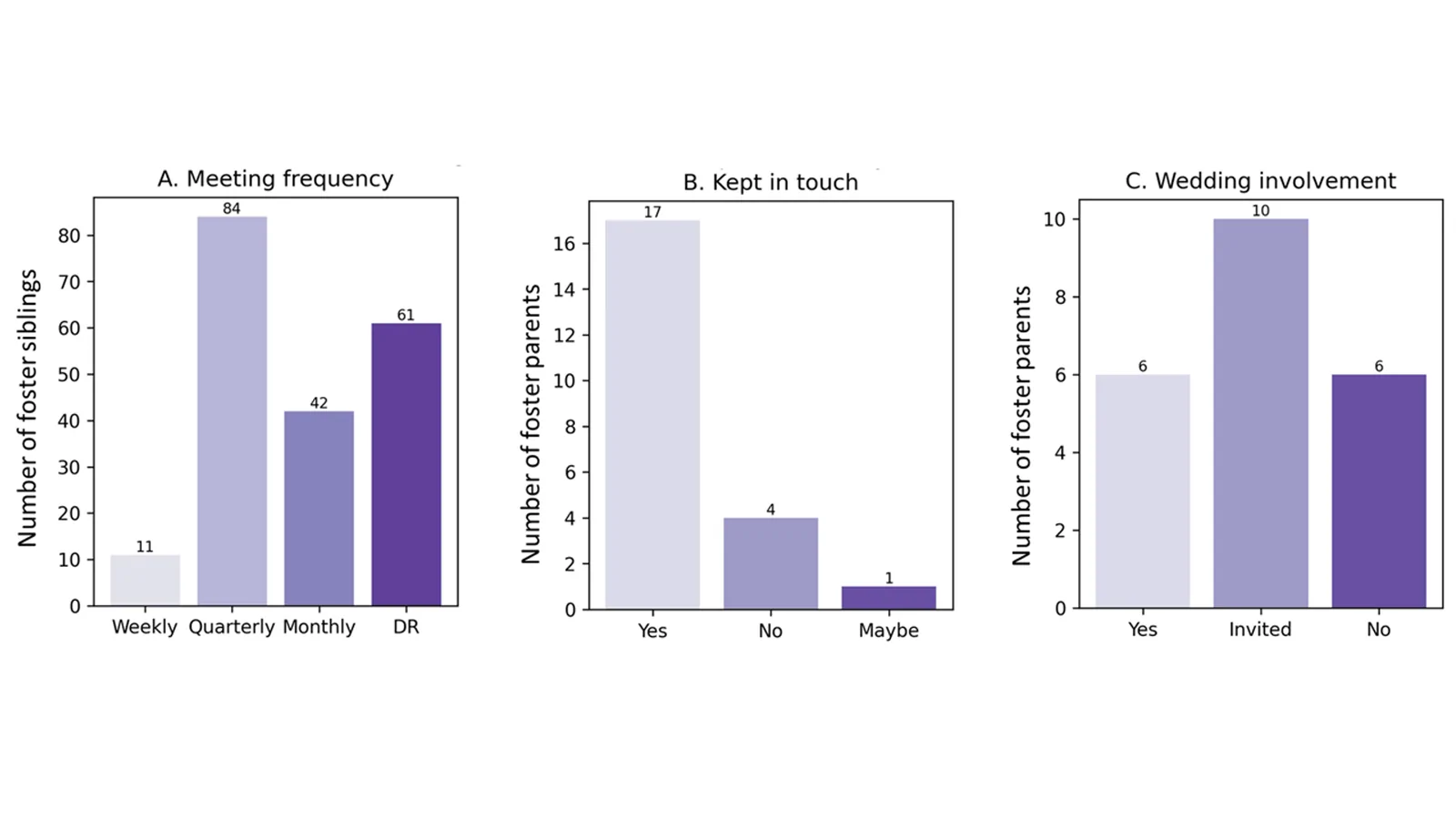
Long-term engagement and relationship continuity. (A) Foster siblings most commonly reported meeting their foster families on a quarterly basis, followed by meetings conducted as required (DR) and monthly interactions, while weekly meetings were less frequent. (B) Most foster parents reported that they remained in contact with their foster siblings even after graduation, indicating the development of enduring mentor–mentee relationships. (C) Foster parents also described continued social involvement with former foster siblings, including invitations to significant life events such as weddings, reflecting the family-like bonds fostered through the program.

A majority of 143 (71.5%) believed that there was a strong potential for replicating the foster family model, although only 36 (19.7% ) felt it was feasible to replicate. Among foster parents, 10 (41.7%) expressed the ability to replicate the model outside CMCL, while 11 (45.8%) were unsuccessful (Figure 6).

**Figure 6:**
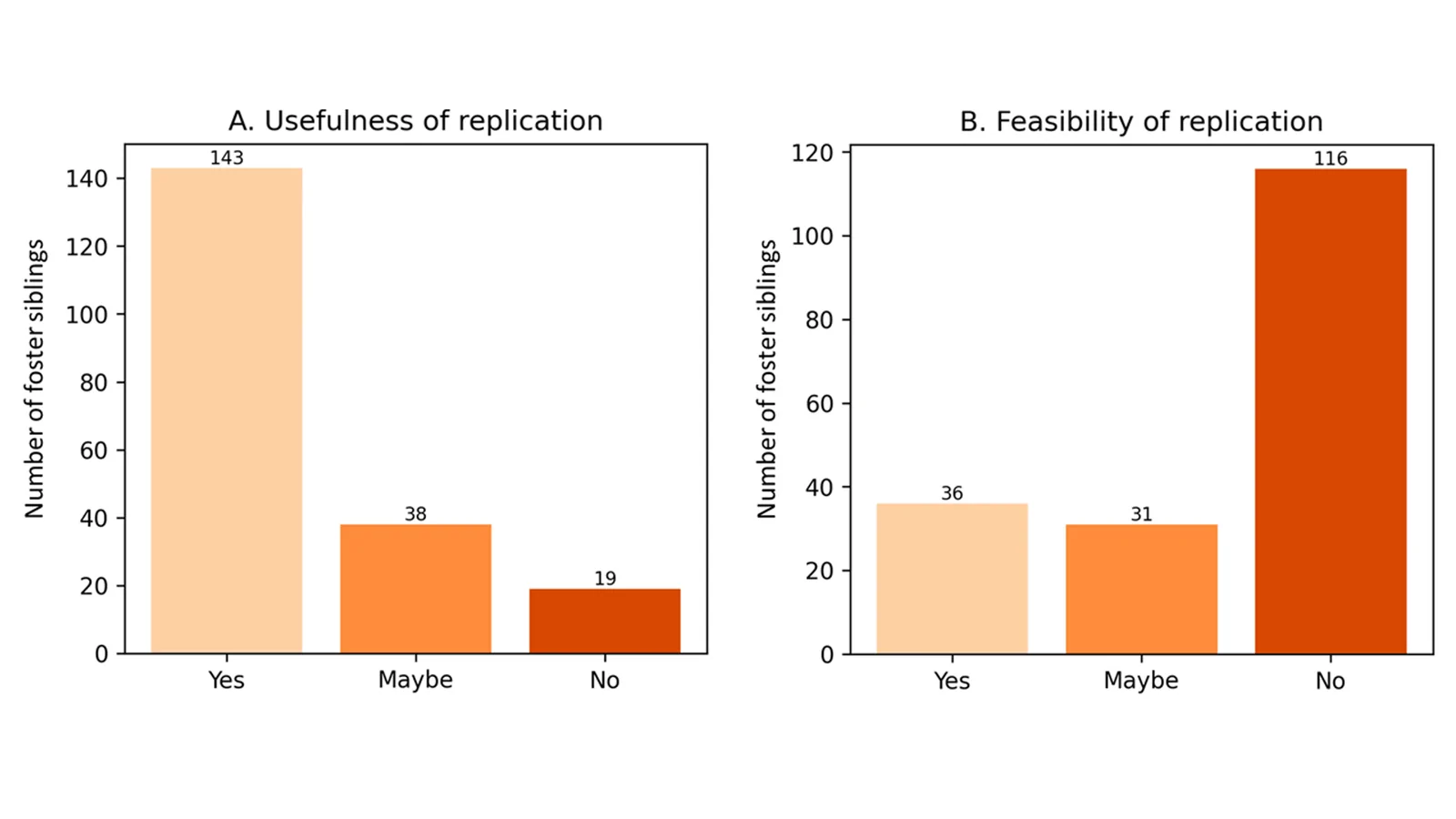
Perceived usefulness and feasibility of replication of the foster sibling intervention. A) Majority of foster siblings perceived the intervention as useful, with 143 participants responding "yes", 38 responding "may be" and 19 responding "no". B) Regarding feasibility of replication , 36 participants responded "yes", 31 responded "may be", and 116 responded "No", indicating that while the intervention was largely considered useful, concerns remained  regarding its practical replication in other settings.

Foster siblings’ perceptions of their foster parents were generally positive. Nearly half ( n = 95, 47.5%) strongly agreed that their foster parent was approachable, and 52 (26.1%) strongly agreed they could share personal problems with them. 39 (25.5%) siblings identified foster parents as a source of support, while 82 (53.6%) identified both foster parents and siblings as sources of support.

The association analysis revealed significant differences between foster siblings and foster parents in key communication variables, including sharing of personal issues, academic performance, and mental health discussions (all p < 0.001), whereas interaction frequency (p = 0.237) and spiritual growth (p = 0.203) did not differ significantly, indicating that qualitative aspects of communication rather than frequency distinguish the groups. These differences are further contextualized by strong demographic contrasts in age, batch, and institutional experience (all p ≤ 0.018), reflecting a generational divide. The correlation heatmap supports these findings by showing strong structural relationships (e.g., age and batch, r = −0.99) and highlights that mentorship effectiveness is driven primarily by interpersonal dynamics, with approachability strongly associated with career guidance (r = 0.68) and mentor influence (r = 0.74), while the overall benefit of interaction remains weakly linked to structural factors. Suggesting that the effectiveness of the foster mentorship program depends less on demographic or interaction frequency factors and more on the quality of engagement, particularly approachability, communication, and relationship (Table 1).

**Table 1:** Association between variables and study factors * indicates statistically significant results (p < 0.05). Exact p-values are reported; values < 0.001 are presented as p < 0.001.

Analysis Group	Variable	Test	Statistic	p-value	Significance
Batch group	Frequency of meeting	Chi-square	27.94	0.001*	Significant
	Share personal issues	Chi-square	1.47	0.688	Not significant
	Share academic performance	Chi-square	10.53	0.309	Not significant
	Spiritual growth	Chi-square	10.83	0.094	Not significant
	IPR	Chi-square	0.65	0.884	Not significant
	Mental health issues	Chi-square	7.86	0.249	Not significant
	Academic failure	Chi-square	1.32	0.725	Not significant
	Benefit of interaction	ANOVA	0.83	0.496	Not significant
	Keep in touch	Chi-square	4.29	0.637	Not significant
	Approachability	ANOVA	2.86	0.011*	Significant
	Sharing personal problems	ANOVA	2.29	0.037*	Significant
	Support	Chi-square	16.82	0.156	Not significant
	Academic challenges	Chi-square	33.79	<0.001*	Significant
	Mental health	Chi-square	21.36	0.045*	Significant
	Discuss academic failure	Chi-square	16.72	0.010*	Significant
	Stress and anxiety	Chi-square	17.56	0.007?*	Significant
	Career decisions	ANOVA	2.36	0.032*	Significant
	Mentor	ANOVA	1.49	0.183	Not significant
Age group	Frequency of meeting	Chi-square	17.08	0.147	Not significant
	Share personal issues	Chi-square	2.38	0.667	Not significant
	Share academic performance	Chi-square	20.33	0.206	Not significant
	Spiritual growth	Chi-square	11.01	0.201	Not significant
	IPR	Chi-square	6.00	0.199	Not significant
	Mental health issues	Chi-square	7.27	0.508	Not significant
	Academic failure	Chi-square	0.56	0.968	Not significant
	Benefit of interaction	ANOVA	0.60	0.666	Not significant
	Keep in touch	Chi-square	12.92	0.115	Not significant
	Approachability	ANOVA	1.08	0.376	Not significant
	Sharing personal problems	ANOVA	1.07	0.385	Not significant
	Support	Chi-square	17.91	0.211	Not significant
	Academic challenges	Chi-square	25.21	0.033*	Significant
	Mental health	Chi-square	14.92	0.384	Not significant
	Discuss academic failure	Chi-square	9.37	0.227	Not significant
	Stress and anxiety	Chi-square	7.59	0.370	Not significant
	Career decisions	ANOVA	1.46	0.183	Not significant
	Mentor	ANOVA	1.11	0.361	Not significant
Sibling vs Parent	Share personal issues	Chi-square	222.00	<0.001*	Significant
	Share academic performance	Chi-square	223.00	<0.001*	Significant
	Spiritual growth	Chi-square	3.19	0.203	Not significant
	Mental health issues	Chi-square	161.00	<0.001*	Significant
	Attend wedding	Chi-square	25.34	<0.001*	Significant
	Replicate	Chi-square	5.94	0.051	Not significant
	Frequency of meeting	Chi-square	4.24	0.237	Not significant
Batch association	Frequency of meeting	Chi-square	158.87	0.006*	Significant
	Share personal issues	Chi-square	353.45	<0.001*	Significant
	Share academic performance	Chi-square	462.10	<0.001*	Significant
	Spiritual growth	Chi-square	96.83	0.073	Not significant
	Mental health issues	Chi-square	305.16	<0.001*	Significant
	Keep in touch	Chi-square	35.82	0.147	Not significant
	Attend wedding	Chi-square	67.56	0.002*	Significant
	Replicate	Chi-square	105.88	0.020*	Significant
	Also foster sibling	Chi-square	98.29	<0.001*	Significant

A qualitative thematic analysis was conducted on participants' responses regarding the strengths and demerits of the FFP, with findings categorized according to the type of foster relationship (foster parent and foster sibling). Analysis of the responses identified several recurring themes that reflected both the positive impact and the challenges of the mentoring experience.

Among the strengths, emotional support emerged as the most prominent theme, with participants describing their foster families as caring, approachable, and dependable sources of comfort during difficult times. Many reported that the warmth, affection, and acceptance they received helped them feel "at home" and provided a strong sense of belonging. Spiritual and academic guidance was another major theme, as foster families frequently offered encouragement, mentorship, and practical advice for coping with the academic and emotional demands of medical training. Participants also valued the sense of community and hospitality, highlighting shared meals, celebrations, recreational activities, and an open-door environment that fostered meaningful interpersonal connections. Additionally, many respondents emphasized that their foster families provided a safe and non-judgmental space where they could openly discuss personal, emotional, and academic concerns, promoting trust, emotional well-being, and resilience.

Despite the overwhelmingly positive experiences, the thematic analysis also revealed several challenges. The most commonly reported concern was inactivity and lack of engagement, with many participants indicating that interactions with their foster families were infrequent or absent, limiting the intended benefits of the program. Time constraints among both foster parents and students emerged as another significant barrier, making it difficult to organize regular meetings and maintain sustained relationships. Some foster parents also identified the financial burden associated with hosting students as a practical limitation. A smaller number of respondents described unrealistic or mismatched expectations, where differences in anticipated roles and responsibilities led to dissatisfaction or strained interactions. Finally, mismatch and interpersonal discomfort were reported in a few cases, with participants feeling that randomly assigned foster relationships lacked compatibility, negatively affecting their overall experience.

Overall, the qualitative findings suggest that the FFP was widely perceived as a valuable source of emotional, social, spiritual, and academic support. The program fostered meaningful interpersonal relationships, encouraged emotional openness, and created opportunities for mentorship and personal growth. However, its effectiveness was influenced by the level of engagement, compatibility between foster families and students, and practical factors such as time availability and financial resources. These findings underscore the importance of active participation, careful mentor-mentee matching, and institutional support to maximize the benefits and sustainability of the program.

## Discussion

CMC has a rich heritage of 132 years and has been privileged to design and run a mentorship program termed the FFP since the 1960's. In one of a kind FFP of our institution, the mentors will be referred to as foster parents, the mentees as foster children, and the mentees allotted to each mentor will be known as foster siblings. Thus, each foster family consists of a foster parent, a foster child, and foster siblings.

This study demonstrates that the participants perceived significant academic, psychosocial, and emotional benefits to medical students, with strong interpersonal relationships and mentorship emerging as key strengths. In our study, the foster parents' profiles were distributed across the older age brackets; 8.3% of them had retired at the time of the study. The lower number of foster parents in our study may be explained by the nature of the FFP, wherein a single foster parent may be associated with and support more than one foster student/sibling. In addition, participation in the study was voluntary, and some foster parents may have been less available or willing to participate compared with foster siblings, who constituted the larger proportion of respondents. This was because the program had been running in our institution for over 5 decades, and the respondents were from different age groups. Also, the mentees in the study profile represented both the undergraduate (49.8%) and post-graduate (50.2%) for the same reason. Since the foster parents had an average of 21.2 years of association with the institution, they have the additional benefit of describing the challenges faced, the positive impact, and the changing nature of the mentorship program running for five decades.

CMC's mentorship program is indeed life-changing and invaluable. As described by Steinmann 2017, it is a continuous relationship between the experienced individuals (mentors) and the students (mentees) which brings about the success of the program. Over the years, the program has been beneficial to the mentees, both in their personal growth and in reaching their highest potential [10]. One of the pointers to the effectiveness of the FFP is that, in our study, over half of the foster parents (58.3%) were themselves foster siblings at one point during their academic training in the institution. Our foster family program laid a strong foundation in strengthening the bonds between the mentors and the mentees, and among the mentees allotted to the same mentor. The foster parents (mentors) and foster children (mentees) continued their relationship beyond their training years in the institution. The foster siblings maintained communication beyond their training period at CMC (67.8%), while 77.3% of the foster parents were also in touch with the mentees after they left the institution. Zhou et al. have described that long-term communication yields positive outcomes for both mentees and mentors, creating a virtuous circle [11]. The need to maintain constant communication and accessibility of the mentor was also highlighted by Zhang et al. [8].

The positive perception amongst the foster family members also highlights the carefully structured guidelines for allocation of the mentor- mentees. The institution allots mentors keeping in mind their areas of expertise, administrative roles, commitment levels, similarity in personalities, and cultural backgrounds. This helps to avoid a mismatch of the mentor-mentees, leading to increased satisfaction with the foster family program [12]. Although a few responses indicated the mentor-mentee mismatch leading to discomfort, this should be explained by the fact that the mentee population admitted to the institution is diverse and representative of students from all four corners of the country, while the faculty (mentor) population is not as diverse. This could be one of the reasons for the several challenges identified in our study. One of the main challenges was a perceived lack of engagement by the respondents because of language barriers and cultural mismatch. The strength of our family foster family program was in alignment with the several studies mentioned in the literature. Emotional support, spiritual and academic guidance were the most important factors highlighting the positive impact of the mentoring experience in our institution. This may be attributed to the informal and family-like structure of the program, which promotes trust, openness, and continuity beyond formal academic settings. Challenges such as time constraints and mentor-mentee mismatch highlight the need for pairing strategies and structural intersection schedules. This has been possible due to the formal structuring of the program at the institutional level, ensuring a healthy organizational climate within the mentoring program, which is one of the key factors defining the success of such a program [13]. The foster family program has administrative support to provide the best mentoring culture, set guidelines related to the program, and even define policies regarding the successful implementation of the program. Mantzourani et al also emphasized the need for a well-designed mentorship program to enhance the mentoring experience [14].

Our study highlights the need to establish strong professional relationships to attain the goals of the mentorship program, as was highlighted by Sheehan et al [15]. As highlighted in our study, providing emotional nurturing, sharing meals and attending weddings of foster siblings/ mentees are indicators of a sense of personal and emotional attachment that goes beyond the normal boundaries of this program's academic engagement and career counseling. These factors also help in self-enhancement by affecting soft skills, as stated by Steinmann et al [16]. Our study emphasizes the need for a clear, well-structured mentorship program, which is affected by the dynamics of the mentor, mentees, and the institution itself, as stated by Stapley et al 2022 [17].

Limitations and Future Directions

This study has several important limitations. First, its retrospective cross-sectional design captures respondents’ perceptions and recollections rather than objective academic, professional, mental-health, or other program outcomes. It therefore cannot establish the effectiveness, impact, or causal effects of the FFP. Second, recruitment used voluntary snowball sampling through alumni networks, email, and social media. Individuals with stronger institutional connections or particularly memorable positive or negative experiences may have been more likely to participate and recruit peers. The absence of a complete sampling frame also prevented calculation of a conventional response rate. These factors may have introduced selection bias and limited the representativeness of the findings. Third, participants spanned alumni cohorts from 1960 to 2015. Recollection of FFP experiences may differ by time since graduation, and the institutional, curricular, social, and demographic contexts of medical education changed substantially during this period. The sample was also weighted toward more recent foster-sibling cohorts, with 44.1% from the 2010s and 23.8% from the 2000s. Consequently, findings should not be interpreted as uniform across all program eras. Although the FFP predates CBME, this study does not evaluate the effect of CBME implementation, which began after the study’s latest graduating cohort. Fourth, only 24 foster parents participated compared with 203 foster siblings. This imbalance limits the precision and stability of foster-parent estimates and makes comparative statistical analyses between roles unreliable. Foster-parent findings should therefore be interpreted descriptively. Finally, the questionnaire was developed for this study and underwent expert content review but did not undergo formal psychometric validation. The results should be interpreted as item-level self-reports rather than precise measures of constructs such as mentor effectiveness, wellbeing, or overall program impact.

Future research should use prospective, cohort-specific designs with predefined outcomes, standardized implementation documentation, validated multi-item measures where appropriate, and recruitment strategies that permit more representative sampling. Evaluation across multiple institutions should precede any recommendation regarding wider transferability of the model

## Conclusions

The FFP was generally described by this self-selected respondent group as a valued source of perceived emotional, social, academic, and relational support. Respondents also identified variable engagement, time limitations, financial burden, and mismatched expectations as important challenges. Because the study relied on retrospective self-reports from a voluntary snowball sample, used a study-specific questionnaire, included highly unequal role-group sizes, and covered a broad span of alumni cohorts, it does not establish program effectiveness, causal impact, or generalizability to other institutions. The findings should be viewed as hypothesis-generating and can inform prospective evaluation and refinement of the FFP within its local context.

## Appendices

Supplementary Appendix 1

Study-specific questionnaire used in the FOSTER study

Participant information and consent

The Foster Family Program (FFP) at Christian Medical College, Ludhiana, is a family-based mentorship programme in which faculty mentors are referred to as foster parents and students are referred to as foster siblings. This questionnaire was designed to describe participants’ retrospective experiences and perceptions of the program, including perceived support, engagement, relationship continuity, strengths, challenges, and views on transferability.

Participation was voluntary. Participants could stop the survey at any point and could choose not to answer individual questions. Responses were anonymised and used only for research purposes. The survey did not assess or establish the effectiveness or causal impact of the programme.

Consent item: “I have read and understood the study information. I voluntarily agree to participate in this survey.” Response options: Yes / No

Role selection: “You are completing this questionnaire as a:” Response options: Foster parent / Foster sibling

The original instrument included role-specific sections, closed-ended questions, Likert-type items, and open-ended questions. The questionnaire was developed for this study and reviewed for content relevance, clarity, comprehensiveness, and appropriateness by an expert panel. It was not intended as a validated psychometric scale.

Section A: Foster-parent questionnaire

Background information

What is your age in years? Response: Numeric entry.

Which MBBS batch did you belong to at CMC Ludhiana? Response: Year of entry or graduation.

What is your current professional role or employment status? Response options: Employed / Retired / Other (specify).

4.     What is your highest professional qualification? Response: Free text.

5.   For how many years were you associated with CMC Ludhiana? Response: Numeric entry.

6.   For how many years did you serve as a foster parent? Response: Numeric entry; enter 0 if less than one year.

7. Before becoming a foster parent, were you a foster sibling as a student? Response options: Yes / No / Do not recall.

Foster-parent experience

1. How often did your foster family meet during the period of your involvement? Response options: Weekly / Monthly / Quarterly / Less often than quarterly / Do not recall.

2.  During your time as a foster parent, did foster siblings feel able to discuss personal concerns with you or the foster family? Response options: Yes / No / Sometimes / Do not know.

3. How often did foster siblings discuss academic performance or academic concerns with the foster family? Response options: Never / Rarely / Occasionally / After professional examinations / Regularly (monthly to quarterly) / Do not recall.

4.     In your view, did the FFP provide opportunities for foster siblings to discuss emotional or mental-health concerns? Response options: Yes / No / Sometimes / Do not know.

5.     Did foster siblings approach you or the foster family after an academic failure or setback? Response options: Yes / No / Sometimes / Do not know.

6.     In your view, did participation in the FFP contribute to foster siblings’ interpersonal connections with peers, seniors, juniors, or faculty? Response options: Yes / No / Uncertain.

7.     In your view, did the FFP support spiritual growth among foster-family members? Response options: Yes / No / Uncertain / Not applicable.

8.     Overall, how beneficial did you find your interactions with foster siblings for understanding student life? Response options: 1 = Not beneficial at all; 2; 3; 4; 5 = Very beneficial.

9.     Do you remain in contact with any foster siblings after they left CMC Ludhiana? Response options: Yes / No / Unsure.

10.  Have you attended the wedding of a former foster sibling? Response options: Yes / No / I was invited but could not attend / Not applicable.

11.  Have you attempted to use a similar mentoring or foster-family approach outside CMC Ludhiana, such as in a workplace or another institution? Response options: Yes / No / Not applicable.

Open-ended questions

1.     What aspects of the Foster Family Program did you perceive as most valuable?

2.     What challenges or limitations did you experience in the Foster Family Program?

3.     What changes would you suggest to improve the Foster Family Program?

Section B: Foster-sibling questionnaire

Background information

1.     What is your age in years? Response: Numeric entry.

2.     Which MBBS batch did you belong to at CMC Ludhiana? Response: Year of entry or graduation.

3.     How many years have passed since you completed your most recent degree at CMC Ludhiana? Response: Numeric entry.

4.     For how many years were you a foster sibling? Response: Numeric entry; enter 0 if less than one year.

5.     Are you currently serving as a foster parent? Response options: Yes / No.

Foster-sibling experience

1.     How often did your foster family meet during your period as a student? Response options: Weekly / Monthly / Quarterly / Less often than quarterly / Do not recall.

2.     To what extent do you agree with the following statement: “My foster parent was approachable when I needed support.” Response options: Strongly disagree / Disagree / Neither agree nor disagree / Agree / Strongly agree.

3.     To what extent do you agree with the following statement: “I felt comfortable discussing personal concerns with my foster family.” Response options: Strongly disagree / Disagree / Neither agree nor disagree / Agree / Strongly agree.

4.     Who was your main source of support within the foster family? Response options: Foster parent(s) / Foster sibling(s) / Both foster parent(s) and foster sibling(s) / None / Other (specify).

5.     During your medical training, did you experience an academic failure or major academic setback? Response options: Yes / No / Prefer not to answer.

6.     If you experienced an academic failure or setback, were you able to discuss it with your foster family? Response options: Yes / No / Partly / Not applicable.

7.     Were you able to discuss academic performance or academic challenges with your foster family? Response options: Yes / No / Partly / Not applicable.

8.     To what extent did the FFP contribute to your interpersonal relationships with peers, seniors, juniors, or faculty? Response options: Not at all / A little / Moderately / Considerably / Very considerably.

9.     Did you perceive the FFP as contributing to your spiritual growth? Response options: Yes / No / Uncertain / Not applicable.

10.  Who did you find most comfortable to approach for emotional or mental-health concerns within the foster family? Response options: Foster parent(s) / Foster sibling(s) / Both / No one / Not applicable / Prefer not to answer.

11.  Did you perceive the foster family as supportive during periods of stress or anxiety? Response options: Yes / No / Partly / Not applicable.

12.  How helpful did you find the foster family in relation to career decisions or choices? Response options: 1 = Not helpful at all; 2; 3; 4; 5 = Very helpful; Not applicable.

13.  To what extent do you agree with the following statement: “My foster parent was an inspiration or role model to me.” Response options: Strongly disagree / Disagree / Neither agree nor disagree / Agree / Strongly agree.

14.  Did you invite your foster parent(s) to your wedding? Response options: Yes / No / I did not marry / Not applicable / Prefer not to answer.

15.  Who do you still keep in contact with from your foster family? Response options: Foster parent(s) only / Foster sibling(s) only / Both foster parent(s) and foster sibling(s) / No one / Other (specify).

16.  Do you believe that selected features of the FFP could be useful in another medical institution or workplace? Response options: Yes / No / Uncertain.

17.  Have you personally attempted to use a similar mentoring or foster-family approach outside CMC Ludhiana? Response options: Yes / No / Not applicable.

Open-ended questions

1.     What aspects of the Foster Family Program did you perceive as most valuable?

2.     What challenges or limitations did you experience in the Foster Family Program?

3.     What changes would you suggest to improve the Foster Family Program?
